# CASE REPORT The Use of Both Antegrade and Retrograde Internal Mammary Vessels in a Folded, Stacked Deep Inferior Epigastric Artery Perforator Flap

**Published:** 2010-04-30

**Authors:** Rodney K. Chan, Wojitec Przylecki, Lifei Guo, Stephanie A. Caterson

**Affiliations:** Division of Plastic Surgery, Department of Surgery, Brigham and Women's Hospital, Boston, MA 02445

## Abstract

**Objective:** Deep inferior epigastric artery perforator (DIEP) flap is an excellent option for breast reconstruction in young and active patients who have a history of chest wall radiation. One drawback, however, is that the entire capacity of abdominal pannus cannot be reliably transferred on a single pedicle. The purpose of this case report is to demonstrate a method of maximizing the volume of reconstruction with a dual-pedicled DIEP flap. **Methods:** A case is reported in which both antegrade and retrograde internal mammary vessels were used as recipient sites for a dual-pedicled, folded, stacked DIEP flap. **Results:** Good flows were observed in both sets of recipient vessels intraoperatively. Postoperative imaging revealed patent vascular anastomoses of both pedicles. At 1-year follow-up, there was no evidence of fat necrosis and a satisfactory aesthetic outcome was achieved. **Conclusion:** To maximize the volume of the reconstructed breast, the entire abdominal pannus can be utilized. The retrograde limb of internal mammary vessels can act as the recipient site for the second pedicle, minimizing donor site morbidity.

Autologous breast reconstruction with a deep inferior epigastric artery perforator (DIEP) flap is an excellent option, especially for young and active patients who have a history of radiation. One drawback, although not unique to DIEP flaps, is that the final volume of reconstruction is limited by the available amount of tissue perfused by 1 vascular pedicle. This is especially problematic in thinner patients with only modest abdominal bulk but who would prefer to maintain the size of their large contralateral breast. Several modifications to the abdominal flaps have been described in order to give the resulting breast mound greater volume, projection, and contour. We describe here our technique of using a dual-pedicled, folded, stacked DIEP flap with the use of both antegrade and retrograde internal mammary recipient vessels.

## CASE REPORT

**Figure 1:**
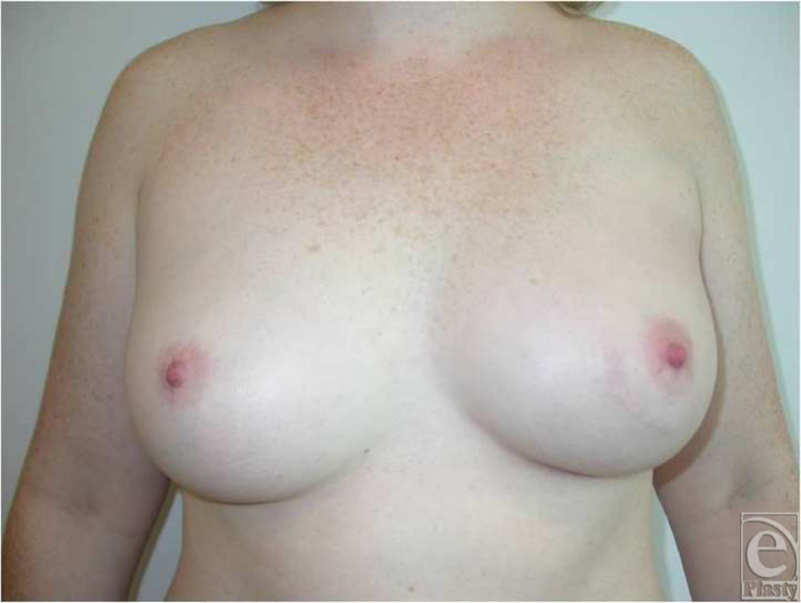
Preoperative frontal view.

**Figure 2:**
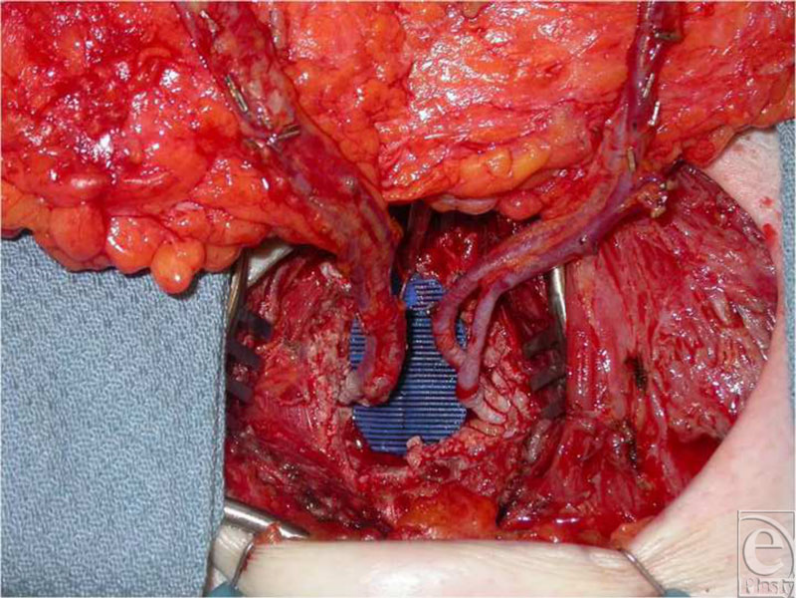
Intraoperative view showing both sets of anastomoses. Vessels to the cutaneous flap are anastomosed to the antegrade mammary vessels (right), and vessels to the buried flap are anastomosed to the retrograde mammary vessels (left).

**Figure 3:**
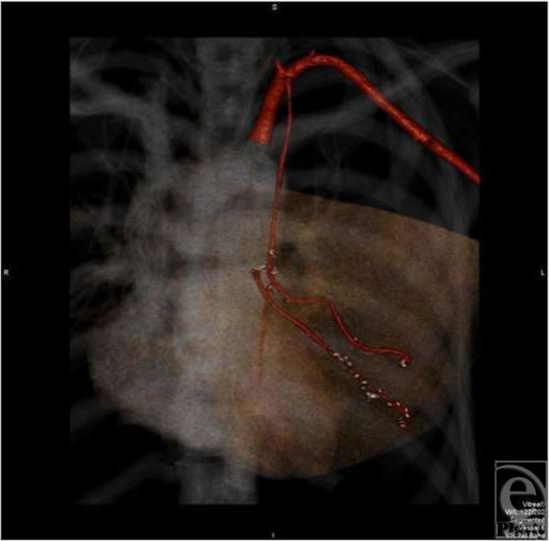
Computed tomographic angiogram performed on postoperative day 5 showing that both antegrade and retrograde arteries are patent.

**Figure 4:**
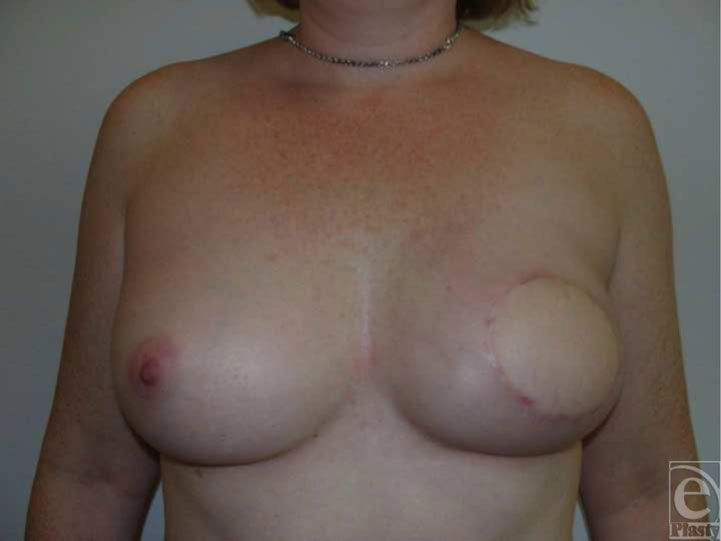
Postoperative view with satisfactory outcome.

A case is presented for a 38-year-old woman with recurrent ductal carcinoma in situ (DCIS) and previous whole-breast irradiation who elected to undergo a total mastectomy with immediate reconstruction. She was an active runner and preferred a DIEP flap reconstruction. Her physical examination revealed only a moderate amount of infraumbilical soft tissue. Half of this abdominal pannus supplied by a single pedicle would not carry enough volume to match her large contralateral breast with significant projection (Fig 1). A large-volume contralateral reduction was not preferable for this patient. A bipedicled, stacked DIEP flap would supply appropriate volume by utilizing all available abdominal donor tissue. Intraoperatively, 2 single perforators were chosen on each hemi-abdomen. The perforators were dissected down to their respective deep inferior epigastric pedicles. The abdominal tissue was transferred as a single unit, without dividing the skin in the midline. After harvesting the abdominal tissue, the flap was folded on itself and microsurgery was performed. One set of deep inferior epigastric vessels was anastomosed to the usual antegrade internal mammary vessels, whereas the other set of vessels was anastomosed to the retrograde internal mammary vessels. Good flows were observed in both recipient arteries. The folded flap was then inset in a stacked manner to maximize volume and projection. All buried skin was de-epithelialized. Keeping the dermis and fat intact between the 2 hemi-abdominal flaps enhanced cross-venous drainage, whereas folding the flap added projection and contour to the inferior pole (Fig 2). Computed tomographic angiogram of the chest on postoperative day 5 was performed incidentally for respiratory concerns and revealed patent vascular anastomoses to both pedicles (Fig 3). At 1-year follow-up, a satisfactory aesthetic outcome with a good size match was achieved without fat necrosis (Fig 4).

## DISCUSSION

The ultimate goal of any breast reconstruction is to provide an aesthetically pleasing mound with appropriate symmetry and contour while minimizing scars. Decision making regarding the choice of technique depends on both the amount of skin deficiency and the amount of 3-dimensional volume losses in comparison with the contralateral breast. Patients with large breast and minimal excess abdominal soft tissue may require the use of all zones of their donor site for appropriate volume. Our use of a dual-pedicled DIEP flap to maximize vascularized tissue transfer involves 2 separate anastomoses, effectively obliterating the poorly vascularized zone II and zone IV. Stacking DIEP flaps also increases projection, whereas folding of the intact de-epithelialized dermis preserves all collateral cross-midline blood flow while enhances inferior pole contour. Retrograde internal mammary artery and vein are adequate to supply and drain the hemi-flap.

Different refinements have been described, especially in transverse rectus abdominis musculo cutaneous flaps (TRAMs) as well as in free TRAMs and DIEPs, to increase the volume of this autologous reconstruction.^1^^-^4 Stacked TRAMs were initially described for radical mastectomy defects in which 2 unipedicled TRAMs are raised and placed atop one another, although many find it difficult to justify sacrificing both rectus muscles for a unilateral reconstruction.^5^ For this reason, a folded, stacked DIEP flap is ideal for volume augmentation with minimal morbidity because of preservation of the abdominal wall musculature.^6^,^7^ The largest experience to date of double-pedicled DIEP flaps comes from Agarwal and Gottlieb,^8^ who performed unilateral reconstruction in 14 patients. Similar to this case, they found the retrograde mammary vein to be useful, although they did not describe the use of the retrograde mammary artery as an inflow vessel.^9^ The retrograde mammary artery was used in this case and proved to adequately supply to at least 1 hemi-flap.

Folded flaps follow a general principle in flap design and inset in which 2 ends of the muscle or skin flaps are brought together, increasing size, as well as improving projection and contour. In this reported case, the fold is positioned inferiorly and has the effect of creating a more projecting inferior pole.

This issue of monitoring the buried portion of the flap is an interesting one. As there is no cutaneous component to the buried flap, physical examination would be unreliable. The use of an implantable Doppler probe to monitor the anastomoses to the buried flap is awkward but possible.^10^ Some authors have intentionally left a cutaneous portion of the buried flap, whereas others have performed the anastomoses in series such that the appearance of the cutaneous flap can be used to monitor the flow to the buried flap.^6^ In this case, because there are still cutaneous connections between the 2 hemi-flaps, there is likely crossover flow that can support each other. The flow to the buried retrograde flap was nonetheless demonstrated on an incidental computed tomographic scan performed in the early postoperative period.

## CONCLUSION

Volume augmentation with DIEP flaps was accomplished in this case by a combination of folding and stacking. A single rib-space donor site was used to supply both flaps, namely, the antegrade and retrograde internal mammary vessels. This technique provides an option for patients who require the full bulk of their abdominal pannus to create enough volume for their reconstructed breasts.
